# Optimising function and well-being in older adults: protocol for an integrated research programme in Aotearoa/New Zealand

**DOI:** 10.1186/s12877-022-02845-7

**Published:** 2022-03-16

**Authors:** Sue Lord, Ruth Teh, Rosie Gibson, Moira Smith, Wendy Wrapson, Murray Thomson, Anna Rolleston, Stephen Neville, Lyn McBain, Silvia Del Din, Lynne Taylor, Nicola Kayes, Andrew Kingston, Rebecca Abey-Nesbit, Ngaire Kerse, On behalf of the AWESSoM Project Team, Heather Allore, Karen Campbell, Stephanie Clare, Judith Davey, Peter Gore, Carolyn Gullery, Carol Jagger, Hamish Jamieson, Sarah Mitchell, Simon Moyes, Kathy Peri, Dan Tautolo

**Affiliations:** 1 School of Clinical Sciences, Faculty of Health & Environmental Sciences, Auckland University of Technology, Akoranga Drive, Northcote, Auckland, 0627 New Zealand; 2 School of Population Health, Faculty of Medical and Health Sciences, University of Auckland, Auckland, New Zealand; 3 School of Health Sciences, Massey University, Wellington, New Zealand; 4 Department of Public Health, University of Otago, Wellington, New Zealand; 5 AUT Public Health and Mental Health Research Institute, Auckland University of Technology, Auckland, New Zealand; 6 Department of Oral Sciences, University of Otago, Dunedin, New Zealand; 7 The Centre for Health, Tauranga, New Zealand; 8 COMPASS Health, Wellington, New Zealand; 9 Clinical Ageing Research Unit, Newcastle University, Newcastle Upon Tyne, UK; 10 Population Health Sciences Institute, Faculty of Medical Sciences, Newcastle University, Newcastle Upon Tyne, UK; 11 Department of Medicine, Canterbury District Health Board, Christchurch, New Zealand

**Keywords:** Older adults, Well-being, Dependency, Function, Māori, Pacific, Co-morbidity, Mixed-methods design

## Abstract

**Background:**

Maintaining independence is of key importance to older people. Ways to enable health strategies, strengthen and support whanāu (family) at the community level are needed. The Ageing Well through Eating, Sleeping, Socialising and Mobility (AWESSOM) programme in Aotearoa/New Zealand (NZ) delivers five integrated studies across different ethnicities and ages to optimise well-being and to reverse the trajectory of functional decline and dependence associated with ageing.

**Methods:**

Well-being, independence and the trajectory of dependence are constructs viewed differently according to ethnicity, age, and socio-cultural circumstance. For each AWESSoM study these constructs are defined and guide study development through collaboration with a wide range of stakeholders, and with reference to current evidence. The Compression of Functional Decline model (CFD) underpins aspects of the programme. Interventions vary to optimise engagement and include a co-developed whānau (family) centred initiative (Ngā Pou o Rongo), the use of a novel LifeCurve™App to support behavioural change, development of health and social initiatives to support Pacific elders, and the use of a comprehensive oral health and cognitive stimulation programme for cohorts in aged residential care. Running parallel to these interventions is analysis of large data sets from primary care providers and national health databases to understand complex multi-morbidities and identify those at risk of adverse outcomes. Themes or target areas of sleep, physical activity, oral health, and social connectedness complement social capital and community integration in a balanced programme involving older people across the ability spectrum.

**Discussion:**

AWESSoM delivers a programme of bespoke yet integrated studies. Outcomes and process analysis from this research will inform about novel approaches to implement relevant, socio-cultural interventions to optimise well-being and health, and to reverse the trajectory of decline experienced with age.

**Trial registration:**

The At-risk cohort study was registered by the Australian New Zealand Clinical Trials registry on 08/12/2021 (Registration number ACTRN 12621001679875).

## Background

**Fig. 1:**
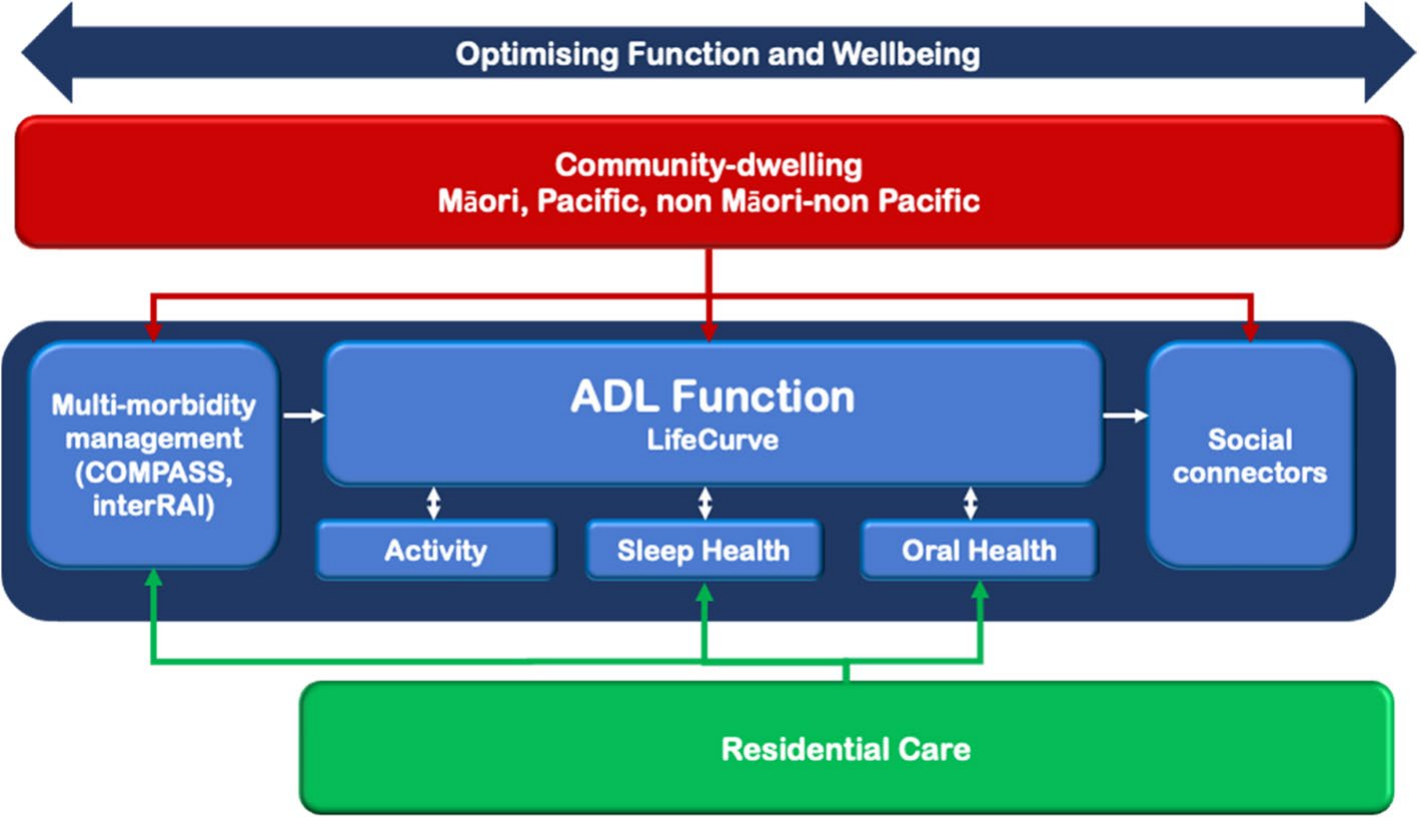
Visual presentation of integrated AWESSoM themes: function & well-being in different populations specifically examining oral health, sleep health, social connection, and physical activity patterns. COMPASS, COMPASS Health (Primary Health Organisation); interRAI, International Residential Assessment

Those aged over 75 years will make up 12.5% (currently 5%) of the New Zealand (NZ) population by 2051, and it is estimated that the proportion over age 85 years will quadruple over the next 20 years. Diversity of the ageing population is also increasing. The majority Pakeha (European) population aged 65 and over will fall in proportions from 88 to 75% from 2013 to 2038 with increases for Asian (5to 16%), Māori (6 to 10%) and Pacific people (2 to 4%) [1, 2]*.* Associated with this is an increase in age-related burden, manifesting as functional decline and attenuated quality of life. Understanding the cause of functional decline and age-related burden is challenging, and although progress has been made the evidence for mitigating risk factors is inconsistent [3, 4]. The need for improved outcomes through many pathways is emphasised, and specific strategies for Māori and Pacific peoples in NZ have been developed. The Ageing Well through Eating, Sleeping, Socialising and Mobility (AWESSoM) programme has two broad aims: firstly to enhance knowledge of risk factors for functional decline especially for populations with scant extant data, and secondly to improve function, health and well-being in older adults. It does so via an inclusive research protocol, drawing on the essential nature of community development, Kaupapa Māori approaches to health, consideration of strengthening of social capital, and broadening of primary health care. The AWESSoM programme delivers five studies that subscribe to an interconnected understanding of function, health and well-being: 1) Māori health and well-being; 2) Pacific elders health and well-being; 3) older community dwellers at risk of functional decline; 4) complex multi-morbidity analyses, and 5) aged residential care. Common themes or target areas for investigation connect these studies and include *sleep, physical activity and mobility, oral health,* and *social connectedness.* These themes are explicitly investigated throughout the programme in all sector groups to a greater or lesser degree, as discussed below (Fig. 1).

### AWESSoM: Māori health & well-being (Nga Pou o Rongo)

Growth in the population of older Māori (indigenous people of Aotearoa/New Zealand) is predicted to be double that expected for non-Māori. Social, economic and environmental factors impact health and political, cultural and historical factors add to these as recognised factors related to health disparities for Māori in NZ. Older Māori are more likely to experience disadvantage and hardship at a level which is three-to-four times that of non-Māori. Higher levels of complex co-morbidities, disability yield differences in longevity between Māori and non-Māori of 8.6 and 7.9 years for men and women respectively [2]. Māori share robust whānau networks; a collective societal responsibility and whakawhānaungatanga (process of establishing relationships, relating well to others). The rate of admission to residential care (termed care homes in this proposal) is lower for Māori than non-Māori [5] and as a result Māori with high levels of disability are cared for at home [2]. Clusters of conditions for Māori differ from those for non-Māori [6] suggesting that ethnic-specific understandings of disease processes are needed.

### AWESSoM: Pacific elders health & well-being (Pacific elders)

Seven percent of NZers identify with at least one Pacific ethnic group, an increase of 11% since 2006 [1]*.* In particular, the Pacific population aged 65 plus is increasing faster than non-Pacific people, and health outcomes for Pacific peoples lag behind those of non-Pacific people in all domains of health [7]. The Healthy Pacific Grandparents (HPG) Study, a participatory action research project developed in collaboration with elder members of Vaka Tautua, a ‘by Pacific for Pacific’ health and social service provider [2] investigated whether the current social and health system environments meet Pacific elders’ needs. Key findings included a recognition by Pacific elders of the importance of taking responsibility for one’s own health and wellbeing but also identified key issues limiting optimal health and well-being and the barriers Pacific elders face in overcoming these. The HPG study also identified a role for technology to enable communication, provide entertainment and leisure options, however limited expertise and lack of exposure impacted on uptake.

### AWESSoM: improving functional decline in older community dwellers (at-risk cohort)

**Fig. 2:**
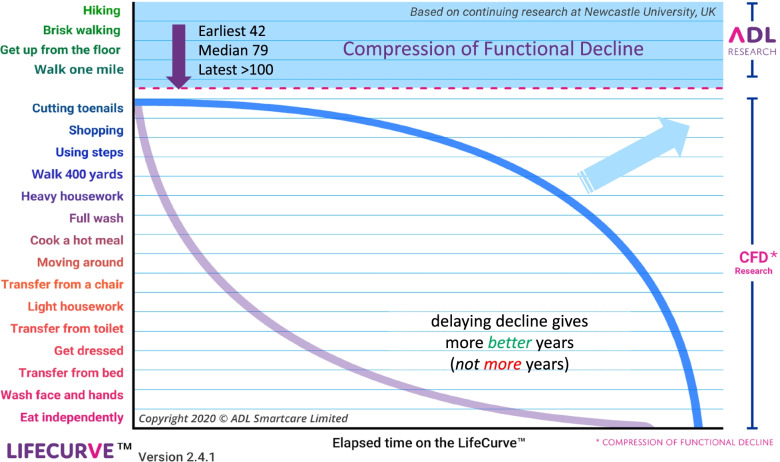
LifeCurve™ hierarchy of functional decline *(reprinted with permission)* [16]

The first target area to be examined in the At-risk cohort is *oral health*, which is a largely unrecognized but major health issue. Demographic and oral epidemiological changes (and associated health issues) among older NZers present an unprecedented situation: poor oral health is considered one of the ‘geriatric giants’ [8]*.* Dental caries remains the major oral health problem in older age, exacerbated by multimorbidity and associated polypharmacy which can compromise saliva flow, leading to higher rates of dental caries, along with problems eating, chewing, speaking and swallowing [9, 10]*.* The second target area is *sleep****,*** which is a problem for over a quarter of older adults [11]. Sleep disturbances are bidirectional, acting both as a precursor to and exacerbating comorbid diseases. For example, sleep disturbances are strongly associated with cardiovascular disease [12]*,* and mental and neurological problems (e.g. depression and/or cognitive impairment), as well as external factors such as social isolation and grief [13]*.* The third area relates to *function*, which manifests as the ability to perform activities of daily living (ADL) [14] in home and community settings (community ADLs are instrumental or extended activities of daily living: IADLs) [15]*.* ADLs and IADLs attenuate in a certain order and operate in a hierarchy of functional domains, described by the Lifecurve hierarchy (see Fig. 2) [17]. The fourth focus area is *physical activity and mobility.* Epidemiological evidence that habitual activity benefits longevity, cardiovascular health and well-being for older people is overwhelming, and this is no less apparent in NZ [18]. Physical activity (or exercise) improves functional status, sleep, health status and life satisfaction for older people [19] and is effective in the treatment of cardiovascular disease, diabetes mellitus, falls and cognitive decline. Despite this, levels of physical activity remain low in older people, and behavioural change strategies are challenging to implement; this is even more so for people with compromised mobility. The final targeted area for the At-risk community cohort is *socialisation.* It is clear that social participation and social support networks are key to long-term outcomes and psychological wellbeing [20]*.* While the exact mechanisms by which social ties and contacts influence psychological morbidity is debated [21], it is clear that social issues contribute to depression and loneliness [22].

### AWESSoM: aged residential care (care home)

Accommodation and health care is provided for our most vulnerable population in aged residential care facilities, termed “care homes” which include rest home, hospital-level and dementia-level care for older people in NZ. Over 60% of NZ and Australian care home residents have memory problems [23] or dementia. AWESSoM takes advantage of NZ’s universal comprehensive geriatric assessment (the international Residential Assessment Instrument, interRAI) [24] to enable identification of individuals with aspects of health relevant to this study, particularly in respect to oral health, sleep, and cognitive decline. Oral health in residential care is a particularly vexing issue, with neither the care sector nor the dental profession prepared for the steadily increasing number of residents entering care with disproportionately high levels of tooth decay, their own teeth, and accompanying risk of aspiration pneumonia [25]*.* Similarly, sleep has been identified as a complex issue within care homes. This is due to an increase of sleep disorders with ageing and cognitive impairment alongside the impact of living and sleeping in a different environment with different schedules and exposures [26]*.*

### AWESSoM: multimorbidity

Multimorbidity may be defined as the presence of two or more chronic conditions with equal predominance [27, 28]*.* Prevalence of multimorbidity is high in older adults, resulting in poor outcomes, polypharmacy [29] and uncertainty about management. People with multimorbidity are 3.5 times more likely to have problems with ADLs and 6 times more likely to have physical function limitations than those without after adjusting for socioeconomic status and depression [30]. Health care costs are 2.5 times higher for those with multimorbidity than for people with a single disease [4]. Guidelines call for individualised treatment, but empirical evidence identifies a lack of understanding of what constitutes individualised care [28]. Patterns of conditions (clusters) may be more relevant for identifying key areas of need and outcomes than a simple count of the number of conditions an individual has [6]*.* For example, cardiovascular clusters lead to the greatest decline in ADLs, while mental health/neurological clusters lead to a decline in IADLs [31]. Inconsistency in the choice of diseases included in studies and statistical methods provides challenges in describing patterns of multimorbidity.

## Methods/design

### Theoretical framework and study design

Models of health, well-being and functional independence underpin the AWESSoM programme. These models vary according to ethnicity, socio-cultural practice, and health/functional status but in essence all lead to an understanding about predictors of functional decline and strategies to mitigate it. Three of the five studies (Māori, At-risk cohort and Care Home studies) trial an intervention. For the Māori study researchers adapted previously trialled participatory action research techniques to develop and refine the intervention [32]. Co-design principles including group discussions, interviews and personal testimonies foster culturally safe practice and demonstrate cultural competence. This practice conforms to Mātauranga Māori - a cultural system of knowledge that reflects a unique Māori worldview. Pacific elders’ data are gathered via individual face-to-face interviews incorporating closed and open-ended questions, and focus group talanoa (conversations) to share views and arrive at common aims and understandings. The compression of functional decline model (CFD) model guides the At-risk study design. The CFD poses that decline in activities of daily living (ADLs) and instrumented activity of daily living (IADLS) is best compressed into the shortest possible time in order to extend the health span and thereby optimize ageing [33]. Decline in ADLs and IADLs occurs in a specific order, as described by the hierarchy of functional loss [17]. Longitudinal studies suggest the trajectory of loss can be reversed or at least remain static. The At-risk study examines this by trialling a novel intervention using a pre-post design. The Care Home and Multi-morbidity studies draw on the National Health Index (NHI) linked interRAI database contains data from standardised assessments of over 300,000 community-dwelling people and 200,000 residential care residents since 2012 with baseline and repeated assessments [24]. The assessment covers function, health conditions, aspects of oral health, nutrition, and mental health as well as indicators of sleep disturbance and function [34]. Finally the Multimorbidity studies also uses a second large extant datasets from primary health, the Compass Health Primary Care Organisation (PCO) dataset (Table 1).

**Table 1:** AWESSoM studies: general characteristics

Study	Key concepts	Aims	Research Design	Outcomes
**Māori older adults** ***P*** Māori community dwelling older adults (age range 55+)	Exercise, nutrition, traditional well-being practices within Te Ao Māori, meditation, stress management, sleep, oral health	Modify and extend a family (whānau-based) lifestyle management strategy (Nga Pou o Rongo) Test scalability & acceptability of Nga Pou o Rongo Examine utility and potential value to Māori of ADL LifeCurve™ App	Conforms to Mātauranga Māori principles: whānau co-design, culturally safe & relevant, local, timely, affirmative, responsive to individual need)	Well-being, physical and social activity & health First in-depth sleep recordings of older Maori and consideration of sleep in contect of whānau support. Acceptability and usability of LifeCurve™ App
**Pacific elders** ***P*** Pacific elders (age range 55+)	Factors associated with ageing well	Investigate how Pacific elders are faring across various dimensions of health Examine utility and potential value of ADL LifeCurve™ App for Pacific elders	Collaborative design with Pacific community partner	Collection of data on Pacific health First representation of sleep health among Pacific elders
**At-risk cohort** ***P*** 300 Community dwelling older adults at risk of functional decline av. age 60 Māori, 75 non-Māori	Innovative technology, health promotion, behavioural change, hierarchy of function, trajectory of functional decline, independent & combined effect of physical activity, sedentary behaviour, socialisation and oral health on function, health & well-being	Adapt a behavioural change tool (LifeCurve™ App) to optimise locality in 2 urban sites Examine utility of and response to the ADL LifeCurve™ App on function, well-being and quality of life in 300 older adults living in the community. Evaluate use of LifeCurve™ App to reverse the trajectory of functional decline in participants Explore the interaction of physical activity, sedentary behaviour, socialisation and oral health on function and well-being.	Mixed methods design. Co-design to modify and develop LifeCurve™AppA for national and local content. Descriptive approach examines: 1) App acceptability, utility & engagment in health promotion activitites; and 2) associations and interactions of key components: physical activity, sedentary behaviour, socialisation, oral health. Pre-post design measures outcomes prior to introduction of LifeCurve™ App and at 12 m. Qualitatively, baseline and post-intervention semi-structured interviews with a diverse group of participants to seek views on App utility. Process evaluation (realist evaluation)	Interactions between sleep, oral health, physical activity, social connectedness, function, health & well-being Successful implementation of social connector model Effect of LifeCurve™ on function, health, well-being. Effect of LifeCurve™ on sleep, oral health, physical activity, social connectedness, function, health & well-being Clinically relevant change of ≥1 point on NEADL. Change ≥2 levels on the LifeCurve hierarchy of function
**Residential care** ***P*** Residents living in aged residential care	Novel interventions to improve oral health, cognition and sleep	Design and implement innovative strategies to improve oral health and cognition including introduction of a dental hygienst and cognitive stimulation theray programme.	Pre-post design with 3 month follow up	Changes in oral health, cognition, sleep and health status Estabishment of long term resources and programmes
**Multi-morbidity** ***P*** Community dwelling older adults, aged care residents (Age 55+; total popluation age 65+)	Patterns of multi-morbidity associated with functional decline and high burden of care.	Discern patterns of multi-morbidity associated with trajectory of dependence and high health services utilization Develop and refine primary care management based on most harmful patterns of multi-morbidity from primary care. Inform the design strategies for Care Home resident based on the most harmful patterns of multi-morbidity	Descriptive design. Multi-morbidity clusters modelled for each setting and for Māori, Pacific, & European using 2 extant data sources: Compass Health PHO and InterRai. Associations will be examined with hospital outcomes and burden of care.	Identify patterns of comorbidities of older adults living in New Zealand. Idenfity groups more likely to enter aged care facilities and others who are at an increased risk of mortality.

*Abbreviations: P* population, *NEADL* Nottingham Extended Activities of Daily Living scale, *PHO* Primary Health Organisation

### Development

The development phase for each study indicates a strong commitment to collaborative and co-participatory research processes (Table 2). For the Māori and Pacific elder studies, this included consultation to ensure culturally appropriate methods to realise AWESSoM aims. For the At-risk cohort it included modification of the LifeCurve™ App to reflect NZ socio-cultural norms and national identity. Local and national resources for the App are embedded within it to ensure that all participants have access to appropriate resources to guide their self-management journey. Embedded themes (oral health, social connectedness, physical activity and sleep) were refined by the research team and integrated to ensure prescription and attainment of common study aims while obtaining novel and relevant findings. The meta-assessment form for non-Māori and non-Pacific participants was trialled and revised accordingly on three occasions over a period of 6 months.

**Table 2:** AWESSoM studies: Collaboration, co-funding, study phases

Study	Patient and Public Involvement (PPI)	Co-funded partnerships	Development	Intervention	Impact & Sustainabilty
**Māori older adults**	Engagement with Māori olders, whānau	N/A	Participation research methods conforming to Mātauranga Māori knowledge and practice. Modification of LifeCurve™ App for Māori.	12 week programme nutrition, sleep, physical activity. Trial of LifeCurve™App.	Potential for broader adoption of intervention within Māori communities; adoption of the App.
**Pacific elders**	Pacific elders	Vaka Tautua (national Pacific health and social service provider)	Engagement with Vaka Tautua	N/A	Potential development of the LifeCurveApp for the Pacific community.
**At-risk cohort**	Māori community; Pacific elders; Age concern	ADL Smartcare BoP DHB Age Concern: national charity representing older adults.	Engagement with Māori and Pacific communities; Engagment with Age Concern and agencies. LifeCurve™ App modification to reflect NZ socio-cultural norms. Identification of broad range of national and regional health services, social & community services to list and describe on the App. Iterarative pilot of the meta-assessment form for use with non-Māori, non-Pacific participants through discussion with each group.	Modification LifeCurve™ App for NZ sociocultural conditions, embed national and local resources on App Recruit participants, train in use of App Self-identification of position on LifeCurve trajectory Use of App for 12 months Concurrent embedded studies physical activity, sedentary behaviour, socialisation, oral health.	Long term adoption of the LifeCurve™ App supported by the BoP DHB. Continued updating of the App to reflect local resources and referral availability.
**Residential care**	Two facilities from a corporate Aged Residential Care organisation.		Engagement with staff and residents to identify key issues regarding oral care, sleep, cognition.	Dential assessments, provision of dental hygienist service. Sleep health assessments. Cognitive stimulation therapy.	Developent of approproate resources for oral care, sleep disturbance, cognitive stimulation
**Profiles of multi-morbidity**	Compass Health: PHO > 500,000 patients enrolled; 2 primary care practices selected for data capture.		N/A	N/A	Identify support needs for clusters of individuals to improve management of co-morbidities and enable independent living for longer.

*Abbreviations*: *ADL* Activities of Daily Living scale, *BoP* Bay of Plenty, *DHB* District Health Board, *PHO* Primary Health Organisation

### Setting, study population and recruitment

Study populations are described in Table 1. All settings are in NZ. Māori, Pacific elders and the At-risk cohort studies are based in community settings, while the Care Home study is sited in two large aged residential care facilities run by the same organisation. The Māori study (Ngā Pou o Rongo) aims to enrol up to 40 whānau using the Manawa Ora Centre for Health (MCH) extensive community networks. Each whānau will be centered around a key individual who is an older person (Māori over 60 years) and comprised of between 2 and 7 whānau members. All participants will sign informed consent forms and attend an introductory whānau hui (meeting). The Pacific Island Grandparents study works with a large Pacific provider in South Auckland to identify people from Samoan, Tongan and Cook Island Maori groups to undertake health assessments and work towards health promotion focused strategies. Included in the At-risk cohort are community dwelling adults (aged at least 60 for Māori and Pacific, and at least 75 for non-Māori) who present with early signs of functional decline as indicated by 1–2 self-identified functional deficits on the LifeCurve™ App trajectory. Excluded from the sample are those who are not able to interact with the App, those lacking capacity to give signed consent, and those living in care homes or in palliative care. The study is located at two convenient study sites. Health services personnel in Tauranga (Bay of Plenty) are members of the AWESSoM research group and Howick (Auckland) is a research site with established research relationships with Easthealth Primary Health Organisation and local stakeholders working with older adults. Both sites participated in the Staying Upright and Eating Well (SUPER) trial, a nutrition study for pre-frail older adults and successfully used general practices to contact adults over 75 years (60 years for Māori) [35]. Older adults from Tauranga and Howick will be contacted and invited to participate using multiple approaches. Older adults will also access the study independently through the web-based platform which is linked to local health services. Other strategies to achieve recruitment targets include a series of planned workshops in both centres, local and national advertisements, medical practices, national organization supports, social media etc. Prior to data collection, all participants will sign informed consent forms compliant with national and ethical regulations. The multimorbidity project uses existing data and matches this with hospitalisations and mortality and aged residential care (ARC) placement data routinely collected.

### Data collection, assessment, intervention

As stated above, for older Māori (Nga Pou o Rongo) Kaupapa Māori co-design research is used for data collection and assessment. Non-pharmacological interventions underpinned by Māori models of health addressing healthy eating, exercise and/or sleep will then be co-designed, incorporating the values, contexts and goals of the whole whānau, and trialled for 12 weeks, concluding with a post programme assessment and completion hui. Whānau will be supported by the research team with contact at fortnightly intervals. Outcome measures will include: Kaupapa Māori quality of life questionnaire, physical activity, functional status, health related quality of life, sleep health and oral health. Whānau will also have the opportunity to develop and include their own outcome measure/s. Sleep is measured objectively using actigraphy among case studies where sleep is identified as a problem from the data collection phase. For the Pacific study, participants undergo a face-to-face assessment to provide essential data on Pacific elders’ wellbeing. Participants in the At-risk study undergo baseline assessment in home, clinical, research, or community settings such as service clubs, community halls, etc. Participants receive training in using the LiveCurve App by the research team, and are then asked to use it for the following 12 months. Support from the research team is available but is not proactively offered in this ‘real-world’ study. App usage will be monitored. There are no specific strategies to enhance adherence, however, this will be evaluated at the end of the trial. All other usual care will continue. There are no specific criteria for discontinuing use of the App because it is a self-monitored, low-risk intervention., After 11 months, participants will be contacted to select a convenient assessment time for the 12 month assessment which is a repeat of all baseline measures. A purposeful sample of App-users seeking diversity on a range of key characteristics (e.g. ethnicity, living situation, gender, baseline characteristics) will be invited to take part in semi-structured interviews at baseline and 12 months to explore their experiences and perspectives of well-being strategies, ageing well, and App use. Care home: oral health will be explored via standardised assessments and the introduction of a dental hygienist for 2 years at two care homes in Wellington region. Simultaneously, cognition and sleep will be measured prior to and after a 7 week trial of Cognitive stimulation therapy and chair yoga using standardised scales and actigraphy sleep monitoring. Interviews with care partners will also be incorporated to better understand the key themes throughout AWESSoM with regards to assessment and management. Assessment and outcome measures for all studies are metrically robust, and identified by expert teams using published evidence, expert experience and research knowledge (summarised in Table 3).

**Table 3:** AWESSoM measures with reference to populations

Characteristic	Measure	Population
Personal characteristics Socioeconomic status	Ethnicity, sex, age, Living arrangement, Pension status, geographical location	Community dwellers, Care home residents, Māori, Pacific
Functional status	NEADL	Community dwellers, Māori, Pacific elders
Cognition	RUDAS MoCA	Community dwellers, Pacific elders Care home residents
Medical history	Menu list of medical diagnoses including: Cardiovascular disease, Chronic Obstructive Pulmonary Disease, Parkinson’s disease, and all relevant diagnoses.	Community dwellers, Māori, Pacific elders
Well-being, Quality of life	SF-36 Physical and mental subscale scores Qual-AD [36] Kaupapa Māori quality of life questionnaire	Community dwellers, Māori Care home residents Māori
Physical performance	Short Physical Performance Battery & Timed Up and Go (TUG) [37].	Community dwellers, Care home residents, Māori, Pacific elders
Habitual activity	7 day accelerometry monitoring of physical activity, sedentary behaviour & gait parameters CHAMPS activity questionnaire	Community dwellers, Māori Community dwellers
Oral health	Oral-health-related quality of life and self-reported oral health (OHIP-14 + Locker item and items on chewing ability), eating assessment tool (EAT-10; dysphagia), oral health assessment tool (OHAT) [38], summated xerostomia inventory (SXI; dry mouth) [39]	Community dwellers, Care home residents, Māori, Pacific elders
Social connectedness	UC UCLA loneliness questionnaire (3 items) [40]; Lubben Social Network Scale [41]	Community dwellers, Pacific elders (UCLA only)
Nutritional health	SCREEN II as a validated (in NZ) nutrition risk screening tool [42].	Community dwellers, Māori, Pacific
Sleep health	Actigraphic sleep monitoring	Community dwellers, care home residents, and Māori identified as ‘problem sleepers’ by self-report and/or standard cut-off scores
	PSQI, rMEQ and the STOP-Bang for sleep disordered breathing [43] Richards-Campbell Sleep Questionnaire [44] Semi-structured sleep interviews	Community dwellers, Māori, Pacific elders (selected outcomes) Care home residents Care home (staff), Māori Māori

*CHAMPS* The Community Health Activities Model Program for Seniors physical activity self-report questionnaire [45], *NEADL* Nottingham Extended Activities of Daily Living Scale [46], *MOCA* Montreal Cognitive Assessment [47], *PSQI* Pittsburgh Sleep Quality Index [48], *Qual-AD* Quality of life in Alzheimer’s Disease [36], *OHIP-14*Oral Health Impact Profile-1 4[49], *rMEQ* Reduced Morning Eveningess Questionnaire [50], *RUDAS* Rowland Universal Dementia Rating Scale, [51] *SF36* Short Form 36 [52], *SXI* Summated Xerostomia Inventory [53]

### Adverse events

All AWESSoM studies are considered low-risk, however a Hazard and Risk Register and Hazard and risk Identification and resolution plan will be developed by the research team who will monitor adverse events 3 monthly. Incident forms will be available to all assessors and any reported incidents will be identified and relevant agencies informed. Any injury linked to the research is covered by NZ’s Accident Compensation Corporation (ACC) scheme which is detailed on the Participant Information Sheet as an ethical requirement.

### Sample size calculation and data analysis

Estimates for sample size are considered formally for the At-risk cohort and the Multimorbidity study. The primary pre-post outcome for the At-risk cohort is functional status, measured by the Nottingham Extended Activities of Daily Living (NEADL). A power calculation based on data from a large longitudinal cohort examining health-status in older Māori and non-Māori (LiLACS NZ) indicated a sample size of 150 participants is required to show a pre-post change of at least 5 points on the NEADL scale which is considered a clinically significant change [46]. The Life Curve™ hierarchy over 12 months will be described for the cohort and patterns of change in response to the App will be modelled. Associations between NEADL, LifeCurve data, independent predictors of health and well-being, and data from oral health, social connectedness, physical activity and sleep will be examined. Objective sleep monitoring will take place for participants with self-identified sleep problems from the Nga Pou o Rongo, At Risk, and Care Home studies wearing the Motionlogger actigraph (Ambulatory Monitoring) (http://www.ambulatory-monitoring.com/motionlogger.html) a small wrist-watch-like device containing an accelerometer for 7 days. Standard sleep variables will be calculated (e.g. sleep timing, efficiency, and duration) as well as non-parametric circadian rhythm variables (inter-daily stability, and intra-daily variability) which are valuable for documenting regulation of sleep. This data will be used alongside subjective measures of sleep and physical and mental health status to better understand the predictors and outcomes of sleep disturbances across the studies and the effect of the App on sleep patterns. Data on App usage (e.g. number of times accessed, duration of use, functions accessed, tasks and activities selected and patterns of use, when and where) will also be evaluated. Information Power [54] will be used to determine sample sufficiency for the qualitative component. Qualitative data will be analysed drawing on reflexive thematic analysis [55]. Data from each time point will be analysed independently before being synthesised as a whole to produce standalone insights regarding the experiences and perspectives of the App and engagement in well-being strategies.

Two separate datasets will be used to examine multimorbidity. A large regional dateset of all primary health care records for over 50,000 patients and separately the national interRAI dataset. The occurrence of multimorbidity (≥2 conditions) and each condition will be described separately for Māori, Pacific, non-Māori/Pacific and by age bands. Using a person-centred approach, agglomerative hierarchical cluster analysis will be used to identify subgroups of patients based on groupings of co-existing conditions. For the clustering algorithm, Ward’s method will be used which sorts individuals into clusters containing a similar profile of conditions [2]. Descriptive statistics will be computed for each of the clusters. Multivariate regression models (linear or log-binomial regression) will be used to examine associations with hospitalisation outcomes. The primary care database will use NHI matching and has worked through the ethical issues to identify the two clusters with the highest impact burden. Similar analysis will be completed separately for inter-RAI data for community dwelling older adults using home care services.

### Data access and confidentiality

All research data will be stored in secure, lockable locations with participants identified only by a numeric ID. All data will have adequate password protection via a secure network drive hosted by the university or health service linked to each study. De-identified information may only be released to the central study coordinating office (University of Auckland) or designee. Data are collected using different platforms including the phone App, mobile tablets, and extant database platforms using specific data collection software and electronic data. Data access will be selectively organized by senior research team members and granted to research personnel according to relevance and research focus. A data monitoring committee has not been established.

### Patient and public involvement

Engagement with older adults, service-users and citizen-led advocacy groups is critical to programme success and viewed as a key driver throughout planning, implementation and evaluation. As reported above, this is particularly pertinent for Māori, Pacific elders and At-risk cohort studies where co-design is a central feature.

### Process evaluation

A process evaluation drawing on a critical realist framework is embedded within the At-risk cohort study to develop a rich and practical understanding of effectiveness with a focus on what works, for who and in what circumstances [56]*.* We will integrate all data sources (e.g. outcome and interview data, App analytics, and demographic details) across time points for each of the participants purposefully sampled to take part in the semi-structured interviews to inform case-specific context-mechanism-outcome (CMO) configurations [57]. CMOs will then be compared, contrasted, and synthesised across cases to produce an in-depth evaluative account of key processes relating to context and implementation to underpin an evidence-based framework for future implementation efforts optimising effectiveness and uptake. No formal economic evaluation is planned.

### Dissemination

A broad approach to dissemination will ensure throughout the 5-year period that stake-holders are informed about protocol development, process and outcomes for each study through hui (meetings), small group discussions, community workshops, conference presentations, seminars, media interviews and journal articles. Authorship eligibility will be considered with respect to guidelines published by and International Committee of Medical Journal Editors (ICMJE), and no professional writers will be used for dissemination purposes.

## Discussion

The AWESSoM programme sets out to understand and influence the predictors of functional decline associated with aging and co-morbidity. Novel aspects to the study include the use of a phone App for those living in the community who are at-risk of functional decline, culturally embedded interventions for Māori and Pasifika people, a focus on oral health and dental hygiene, and analysis of co-morbidity data from two disparate populations. Results from this diverse but integrated programme will inform older adults and their carers, public sector groups, and health, social and community services about ageing, function, and well-being.

## Data Availability

Individual participant data collected during the trial after de-idenfiication will be available upon request to Professor Ngaire Kerse, University of Auckland, NZ, (n.kerse@auckland.ac.nz). Some restrictions are likely to be necessary given the sensitivity of Māori and Pacific data in particular. Access to data does not extend to the public. Requests for the full study protocol may also be submitted to the Professor Ngaire Kerse.

## Abbreviations

ACC: Accident Compensation Corporation
ADL: Activities of daily living
ARC: Aged Residential Care
AWESSoM: Ageing Well through eating, sleeping, socialising and mobility
CFD: Compression of functional decline
CMO: Context-mechanism-outcome
HPG: Healthy Pacific Grandparents
IADL: Instrumented activities of daily living
interRAI: International Resident Assessment Instrument
NEADL: Nottingham extended activities of daily living
NHI: National Health Index
NZ: New Zealand
SUPER: Staying Upright and Eating Well
